# Diagnostic characteristics and prognoses of primary-care patients referred for clinical exercise testing: a prospective observational study

**DOI:** 10.1186/1471-2296-15-71

**Published:** 2014-04-18

**Authors:** Gunnar Nilsson, Thomas Mooe, Hans Stenlund, Eva Samuelsson

**Affiliations:** 1 Department of Public Health and Clinical Medicine, Umeå University, Umeå, Sweden; 2 Department of Public Health and Clinical Medicine, Unit of Clinical Research Centre – Östersund, Umeå University, Umeå, Sweden

**Keywords:** Angina pectoris, Chest pain, Electrocardiography, Exercise test, Myocardial infarction, Myocardial ischemia, Predictive value of tests, Primary health care, Prognosis, Self assessment

## Abstract

**Background:**

Evaluation of angina symptoms in primary care often includes clinical exercise testing. We sought to identify clinical characteristics that predicted the outcome of exercise testing and to describe the occurrence of cardiovascular events during follow-up.

**Methods:**

This study followed patients referred to exercise testing for suspected coronary disease by general practitioners in the County of Jämtland, Sweden (enrolment, 25 months from February 2010). Patient characteristics were registered by pre-test questionnaire. Exercise tests were performed with a bicycle ergometer, a 12-lead electrocardiogram, and validated scales for scoring angina symptoms. Exercise tests were classified as positive (ST-segment depression >1 mm and chest pain indicative of angina), non-conclusive (ST depression or chest pain), or negative. Odds ratios (ORs) for exercise-test outcome were calculated with a bivariate logistic model adjusted for age, sex, systolic blood pressure, and previous cardiovascular events. Cardiovascular events (unstable angina, myocardial infarctions, decisions on revascularization, cardiovascular death, and recurrent angina in primary care) were recorded within six months. A probability cut-off of 10% was used to detect cardiovascular events in relation to the predicted test outcome.

**Results:**

We enrolled 865 patients (mean age 63.5 years, 50.6% men); 6.4% of patients had a positive test, 75.5% were negative, 16.4% were non-conclusive, and 1.7% were not assessable. Positive or non-conclusive test results were predicted by exertional chest pain (OR 2.46, 95% confidence interval (CI) 1.69-3.59), a pathologic ST-T segment on resting electrocardiogram (OR 2.29, 95% CI 1.44-3.63), angina according to the patient (OR 1.70, 95% CI 1.13-2.55), and medication for dyslipidaemia (OR 1.51, 95% CI 1.02-2.23). During follow-up, cardiovascular events occurred in 8% of all patients and 4% were referred to revascularization. Cardiovascular events occurred in 52.7%, 18.3%, and 2% of patients with positive, non-conclusive, or negative tests, respectively. The model predicted 67/69 patients with a cardiovascular event.

**Conclusions:**

Clinical characteristics can be used to predict exercise test outcome. Primary care patients with a negative exercise test have a very low risk of cardiovascular events, within six months. A predictive model based on clinical characteristics can be used to refine the identification of low-risk patients.

## Background

Ischaemic heart disease (IHD) is important to consider in any patient with chest pain or discomfort in the chest [1-6]. Visits due to chest pain account for 0.7-4% of all consultations in primary care [7-10]. The percentage of IHD among patients with chest pain is dependent on the setting [11]. In studies from primary care, 8-18% of all chest-pain patients are diagnosed with heart disease [7-10,12]. Further diagnostic testing is often needed, depending on assumptions of diagnostic probability.

For evaluating chest pain in non-emergency cases, the clinical exercise test has been validated for patients in the primary-care setting [13]. The exercise test, which in Sweden is conducted as a bicycle test with registration of chest pain symptoms and a 12-lead electrocardiogram (ECG), is available by referral from general practitioners (GPs) [14,15]. For diagnosing coronary disease, the exercise test has been criticized for having lower sensitivity and specificity than other non-invasive tests [1,5,16], such as 64-slice computed tomography coronary angiography [17], magnetic resonance imaging techniques [18], dobutamin stress echocardiography [19], or myocardial perfusion scintigraphy with single-photon emission computed tomography [19]. These non-invasive tests are not validated for use in low-prevalence populations, and their availability may be restricted.

In a meta-analysis of studies on magnetic resonance imaging of coronary artery disease, the disease prevalence varied from 57.4% to 70.5% [18]. In the meta-analysis of studies on 64-slice computed tomography coronary angiography, the median prevalence of significant coronary artery disease (≥50% stenosis) was 58% (range 23% to 96%) [17], which is far higher than the prevalence reported by chest-pain studies conducted in primary care [7-10,12]. Frequent diagnoses in patients with chest pain symptoms include chest-wall syndromes, gastro-oesophageal reflux disease, and panic disorder [12,20,21].

The risk of unnecessary advanced investigations of low-risk patients needs attention [22] because such investigations increase total expenditures and may be harmful to the patient. An exercise test is therefore still a diagnostic option for patients with pain or discomfort in the chest in the primary-care setting [13,23]. A structured pre-test work-up using clinical characteristics may be helpful to decide whether an exercise test is worthwhile. Several reports on IHD have focused on chest pain as the main symptom [7,8,10,24-26]. This approach has limitations, since significant symptoms may be described in terms of tightness, discomfort, or indigestion [3]. In this study we sought to recruit all patients referred to exercise testing from a GP due to a suspicion of IHD, irrespective of the initial symptoms. Our primary objective was to identify clinical characteristics that predict the outcome of exercise tests. A second objective was to describe the occurrence of cardiovascular events within six months after testing and to relate these occurrences to test results.

## Methods

This investigation was a prospective observational study of patients referred to exercise testing by GPs.

### Setting and participants

Enrolment occurred from 28 primary-care centres in the County of Jämtland, Sweden. In 2012, 59 485 inhabitants were living in the central municipality of Östersund (47% of the total population), and 66 716 inhabitants were living in one of the seven rural municipalities within the County of Jämtlands län; data provided from Statistics Sweden. All patients with suspected angina were cared for by GPs, who referred patients to exercise testing at the department of clinical physiology, Östersund hospital. The central hospital in Östersund was the only hospital that received referrals from GPs within the study period. The GPs served at primary care centres run by the county council, or centres with contracts for primary care, within the county council of Jämtlands län. There were no other external providers of primary care that referred patients to exercise tests, within the studied county. The period of enrolment was from February 2010 until February 2012. All patients with suspected angina were cared for by GPs, who referred patients to exercise testing at the department of clinical physiology, Östersund hospital. Patients were enrolled if they were at least 20 years old and had consulted for symptoms warranting an exercise test, as determined by a GP. Patients unable to conduct an exercise test or referred for reasons other than a possible IHD were excluded.

### Data sources and measurement

Patient ages were determined by the date of the exercise test. Blood pressure was measured as supine blood pressure with a sphygmomanometer before the exercise test, and during the test with a sphygmomanometer and a Doppler probe placed over the radial artery, to facilitate blood pressure measurements under ongoing exercise.

A pre-test questionnaire addressed present medication, smoking habits, past medical history, chest-pain symptoms, educational level, and one question on angina diagnosis according to the patient’s opinion: “In your own opinion, do you have angina pectoris?” This questionnaire was mailed to patients along with the notice for exercise testing. Questionnaires were collected by nursing staff before the exercise test. The three questions on chest pain were: “Do you ever have chest pain or discomfort in the chest?”; “Do you have chest pain walking at an ordinary pace on the level?”; and “Do you have chest pain walking uphill or in a hurry?” These questions were previously used as a part of the Rose angina questionnaire [27-29]. Questions on present medication and past medical history were presented with the fixed alternative answers of yes or no.

### Exercise test procedure

The exercise tests were conducted by nursing staff and physicians trained in clinical physiology. A 12-lead resting ECG was registered before the exercise test. The ECG recordings were classified by the physician responsible for the test according to the Minnesota Code guidelines [30,31]. The exact criteria for evaluating Q wave and ST-T segment pathology are extensive; therefore, we provide these criteria in Additional file 1. A pathological Q wave refers to the Minnesota Code Q or QS, items I, 1. Exercise tests were conducted with a bicycle ergometer according to national guidelines [14,15]. The initial workload was 30–50 W, considering the patient’s age, sex, and physical condition, with the aim that the patient should exercise for 6–10 minutes. The workload was increased by 10–20 W per minute depending on the initial workload. We used a Cardiolex EC Sense for ECG recordings, and a Rodby RE990 ergometer bicycle.

A 12-lead ECG with computer-assisted reading of mean ST-segment depression was registered during and one and four minutes after exercise. Visual assessment of the ECG recording was possible during the entire test. Systolic blood pressure, respiratory rate, symptoms of chest pain, and level of exertion were registered every two minutes (blood pressure) to every sixth minutes of exercise, until the end of the test. During the exercise test, chest-pain symptoms and perceived exertion were registered according to validated rating scales [32]. Patients were monitored until four minutes after exercise. The referring GP received a statement of the test results and held future responsibility for patient care.

### Classification of exercise tests

The physician responsible for the test classified the exercise tests as positive, non-conclusive, negative, or non-assessable. A positive exercise test was defined as a horizontal or down-sloping depression of the ST segment >0.1 mV at 60 ms after the inflection point between the QRS and ST segments (J point) and chest pain suggestive of angina during the test. Non-conclusive tests were characterized by chest-pain symptoms or ST-segment depression during the test. Negative tests involved neither chest pain nor ST-segment depression during the test. Exercise tests in which the ST segment not could be assessed due to left bundle branch block, pacemaker, or digitalis medication were classified as non-assessable.

### Cardiovascular events

Diagnoses of unstable angina or myocardial infarction, cardiovascular death, recurrent angina in primary care, and decisions on revascularization were recorded during the 6-month follow-up with the electronic medical records system. Patients with a decision on revascularization were followed with respect to the date and type of procedure performed.

A myocardial infarction was defined according to the universal definition [33]. Cardiovascular death was registered when the cause of death was sudden cardiac death, myocardial infarction, or congestive heart failure. Data on causes of death were provided from the Swedish Registry of Causes of Death.

### Statistical methods

Baseline characteristics are presented as means and proportions. The Student’s *t*-test or the chi-squared test was used for group comparisons as appropriate. All patients, except 15 patients with non-assessable tests, were included in the statistical analyses. Univariate logistic regression analyses were performed to identify the clinical variables significantly associated with a positive or inconclusive outcome of exercise testing. These variables were entered into a multivariable logistic model that was reduced via stepwise exclusion of the least-significant variable until all variables were significant. Significant variables and variables assessed to be of clinical importance were retained in the final model. Results from the logistic regression analyses are presented as odds ratios (ORs) with 95% confidence intervals (CIs). Clinical variables that remained significant in the final model were used, one-by-one or in combination, to evaluate their ability to predict exercise-test outcome. The final model was used to calculate individual probabilities for a positive or inconclusive outcome of exercise testing. A probability cut-off of 10% was used to detect cardiovascular events in relation to the predicted outcome of the test. The model was evaluated using Nagelkerke R2, Hosmer-Lemeshow test and a comparison between analytic and bootstrapped standard errors of coefficients. Influential individuals were identified using the Leverage statistic and DfBeta values. The bootstrapping was based on random samples from 865 individuals selected with replacement. In total, 100 replicates were performed. Bootstrapping was performed with Stata (version 12). The diagnostic characteristics for exercise-test outcome were calculated with the software WINPEPI, version 11.26 [34]. All other analyses were performed with IBM SPSS (version 20). The level of significance was set to p < 0.05.

### Ethical approval

Approval was obtained from the Regional Ethical review Board at Umeå University. All patients in the study provided written informed consent.

## Results

**Figure 1:**
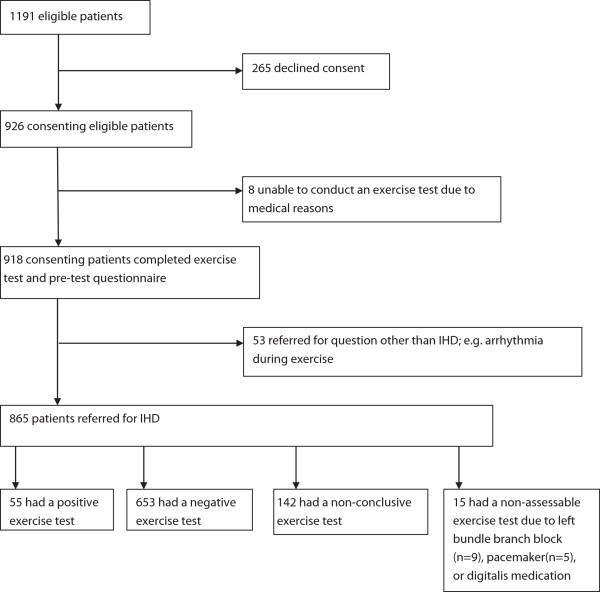
Study profile of participant recruitment and outcomes according to classification of exercise tests.

Of 1191 patients referred for exercise testing, 926 agreed to participate and answered the questionnaire. The study group consisted of the 865 patients, mean age 63.5 years (50.6% men), able to perform an exercise test, that were referred due to suspicion of IHD. Of these, 55 patients (6.4%) had a positive exercise test, 653 (75.5%) were negative, 142 (16.4%) had non-conclusive tests and 15 patients (1.7%) were not assessable (Figure 1).

More than half of the patients were ex-smokers or current smokers. Previous cardiovascular events were more common among men, with 21.3% of men and 11.2% of women associated with at least one previous cardiovascular event. Cardiovascular risk factors were abundant, with more men on medication for dyslipidaemia. Women reported chest-pain symptoms more frequently than men (76.5% vs. 63.8%, respectively, reporting ever experiencing chest pain or discomfort in the chest), but exertional chest pain was equally frequent in men and women (Table 1).

**Table 1:** Characteristics of study participants (n = 865)

	**Total n = 865**	**Men n = 438 (50.6%)**	**Women n = 427 (49.4%)**	**p**
**Age, mean (SD)**	63 (12)	63 (12)	64 (11)	0.030
**University or college degree**	134 (17.3%)	50/394 (12.7%)	84/380 (22.1%)	0.001
**Smoker, current or previous**	455 (52.9%)	245/436 (56.2%)	210/424 (49.5%)	0.050
**Past medical history**				
Myocardial infarction	77 (9.0%)	57/434 (13.1%)	20/424 (4.7%)	<0.001
Revascularization	75 (8.7%)	60/436 (13.8%)	15/426 (3.5%)	<0.001
Stroke or TIA	51 (6.0%)	27/433 (6.2%)	24/422 (5.7%)	0.735
Previous cardiovascular event	139 (16.3%)	92/432 (21.3%)	47/420 (11.2%)	<0.001
**Present conditions**				
Hypertension, medication for	464 (54.2%)	248/435 (57%)	216/421 (51.3%)	0.094
Diabetes mellitus, treatment for	96 (11.2%)	56/434 (12.9%)	40/423 (9.5%)	0.110
Dyslipidaemia, medication for	241 (28.2%)	144/433 (33.3%)	97/421 (23.0%)	0.001
Congestive heart failure, medication for	104 (12.4%)	58/424 (13.7%)	46/414 (11.1%)	0.260
Claudication	48 (5.7%)	25/430 (5.8%)	23/407 (5.7%)	0.919
**Chest pain symptoms**				
Ever have chest pain or discomfort in the chest	587 (70.0%)	272/426 (63.8%)	315/412 (76.5%)	<0.001
Chest pain walking at an ordinary pace on the level	124 (14.7%)	59/431 (13.7%)	65/412 (15.8%)	0.392
Chest pain walking uphill or in a hurry	408 (48.7%)	198/427 (46.4%)	210/411 (51.1%)	0.171
**Angina diagnosis according to patient’s opinion**	211 (26.4%)	111/404 (27.5%)	100/396 (25.3%)	0.476
**Systolic blood pressure (mm Hg), mean (SD)**	147 (19)	148 (19)	147 (20)	0.503
**Diastolic blood pressure (mm Hg), mean (SD)**	84 (10)	85 (10)	84 (10)	0.043
**Resting ECG**				
Normal resting ECG	639 (73.9%)	303/438 (69.2%)	336/427 (78.7%)	0.001
Atrial fibrillation	38 (4.4%)	27/438 (6.2%)	11/427 (2.6%)	0.010
Atrioventricular block	23 (2.7%)	17/438 (3.9%)	6/427 (1.4%)	0.024
Signs of myocardial scarring	73 (8.4%)	45/438 (10.3%)	28/427 (6.6%)	0.049
Pathologic ST-T segment on resting ECG	106 (12.3%)	59/438 (13.5%)	47/427 (11.0%)	0.269

SD: standard deviation. TIA: transitory ischaemic attack. ECG: electrocardiogram. Cardiovascular event: myocardial infarction, revascularization, stroke, or TIA. Myocardial scarring: pathologic Q-wave or altered R-wave progression compared to previous ECG.

Men more often had pathological findings on resting ECG, but a normal ECG was the most common result (73.9% of patients; Table 1). The percentage of positive exercise tests was 8.2% among men and 4.4% among women (p = 0.025). The percentage of negative tests (men 74.2%, women 76.8%) and non-conclusive tests (men 16.0%, women 16.9%) was without significant sex difference. The 142 non-conclusive patients had significant ST-depression in 89 cases (51% men) and chest pain suggestive of angina during the test in 53 cases (47% men).

### Diagnostic characteristics

Patients with a positive/non-conclusive exercise test were older and had higher systolic blood pressure than patients with a negative test (Table 2). Previous cardiovascular events, medication for hypertension and dyslipidaemia, exertional chest pain, and angina diagnosis according to the patient’s opinion were all significantly more common in the positive/non-conclusive group. A normal resting ECG was more frequent among patients with a negative exercise test (Table 2). Chest pain on exertion (walking uphill or in a hurry; OR 2.46, 95% CI 1.69-3.59), pathologic ST-T segment on resting ECG (OR 2.29, 95% CI 1.44-3.63), angina according to the patient’s opinion (OR 1.70, 95% CI 1.13-2.55), and medication for dyslipidaemia (OR 1.51, 95% CI 1.02-2.23) were predictors of exercise-test outcome after adjustment for age, systolic blood pressure, previous cardiovascular events, and sex (Table 3).

**Table 2:** Characteristics of participants with positive/non-conclusive or negative test (n= 850)

	**Exercise test**		
	Positive/non-conclusive*	Negative**	**p**
**Patients**	n=197 (23.2%)	n=653 (76.8%)	
**Age, mean (SD)**	66 (11)	62 (12)	<0.001
**Men**	106 (53.8%)	325 (49.8%)	0.321
**Smoker, current or previous**	108 (54.8%)	342 (52.8%)	0.615
**Past medical history**			
Myocardial infarction	25 (12.8%)	49 (7.6%)	0.024
Revascularization	30 (15.2%)	44 (6.8%)	<0.001
Stroke or TIA	16 (8.1%)	35 (5.4%)	0.171
Previous cardiovascular event	47 (24.0%)	88 (13.7%)	0.001
**Present conditions**			
Hypertension, medication for	120 (61.5%)	332 (51.4%)	0.013
Diabetes mellitus, treatment for	24 (12.2%)	69 (10.7%)	0.541
Dyslipidaemia, medication for	78 (40.0%)	157 (24.4%)	<0.001
Congestive heart failure, medication for	30 (15.6%)	70 (11.1%)	0.094
Claudication	14 (7.4%)	33 (5.2%)	0.247
**Chest pain symptoms**			
Ever have chest pain or discomfort in the chest	138 (72.3%)	436 (69.0%)	0.390
Chest pain walking at an ordinary pace on the level	50 (26.0%)	69 (10.8%)	<0.001
Chest pain walking uphill or in a hurry	134 (69.1%)	264 (42.0%)	<0.001
**Angina diagnosis according to patient’s opinion**	80 (43.2%)	126 (20.9%)	<0.001
**Systolic blood pressure (mm Hg), mean (SD)**	151 (21)	146 (19)	0.001
**Resting ECG**			
Normal resting ECG	132 (67.0%)	507 (77.6%)	0.003
Atrial fibrillation	14 (7.1%)	22 (3.4%)	0.026
Atrioventricular block	4 (2.0%)	19 (2.9%)	0.507
Signs of myocardial scarring	17 (8.6%)	55 (8.4%)	0.927
Pathologic ST-T segment on resting ECG	42 (21.3%)	62 (9.5%)	<0.001

*Positive exercise test: angina symptoms and depression of ST segment >0.1 mV during the test. Non-conclusive test: angina or ST depression. **Negative test: neither angina nor ST depression. SD: standard deviation. TIA: transitory ischaemic attack. ECG: electrocardiogram. Cardiovascular event: myocardial infarction, revascularization, stroke, or TIA. Myocardial scarring: pathologic Q-wave or altered R-wave progression compared to previous ECG.

**Table 3:** Crude and adjusted ORs for a positive/non-conclusive exercise test (n=850)

**Characteristic**	**Crude OR (95% CI)**	**Adjusted OR (95% CI)**
Pathologic ST-T segment on resting ECG	2.58 (1.68-3.97)	2.29 (1.44-3.63)
Angina diagnosis according to patient’s opinion	2.88 (2.03-4.09)	1.70 (1.13-2.55)
Chest pain walking uphill or in a hurry	3.09 (2.19-4.35)	2.46 (1.69-3.59)
Age in years	1.03 (1.01-1.04)	1.01 (0.99-1.03)
Systolic blood pressure in mm Hg	1.01 (1.01-1.02)	1.01 (1.00-1.02)
Dyslipidaemia, medication for	2.07 (1.47-2.90)	1.51 (1.02-2.23)
Previous cardiovascular event	1.98 (1.33-2.95)	1.02 (0.63-1.64)
Male sex	1.18 (0.86-1.62)	1.16 (0.82-1.64)

ORs for a pathologic ST-T segment in the resting ECG, angina according to the patient’s subjective assessment, and exertional chest pain, were adjusted for age, systolic blood pressure, dyslipidaemia, previous cardiovascular events, and male sex. OR: odds ratio. CI: confidence interval. ECG: electrocardiogram. TIA: transitory ischaemic attack. Cardiovascular event: myocardial infarction, revascularization, stroke, or TIA. Positive exercise test: angina symptoms and depression of ST segment >0.1 mV during the test. Non-conclusive test: angina or ST depression.

The predictive characteristics of exertional chest pain, pathologic ST-T segment on resting ECG, and angina according to the patient’s opinion were examined for diagnostic accuracy, separately (Table 4) and in combination (Table 5). Medication for dyslipidaemia was not used as a predicting variable in the composite model because it was dependent on the physicians’ prescription habits and because measurements of serum cholesterol levels were not available before treatment. The pre-test probability (23.2%) was the percentage of positive and non-conclusive exercise tests (n = 197) out of all assessable tests (n = 850). The positive predictive values and likelihood ratios for a positive test (LR+) for the separate characteristics (Table 4) increased in the composite model (Table 5). A combination of exertional chest pain with a finding of pathologic ST-T segment on resting ECG or with angina according to the patient’s opinion decreased the number of patients per positive/non-conclusive case from 3 to 1.7 or 2.2, respectively (Tables 4 and 5).

**Table 4:** Diagnostic characteristics of exercise test outcome (positive/non-conclusive tests)

**Characteristic**	Exertional chest pain*	Pathologic ST-T segment**	**Angina diagnosis according to the patient’s opinion**^**†**^
	**(95% CI)**	**(95% CI)**	**(95% CI)**
Number of patients with characteristic	134	42	80
Sensitivity	69.1% (62.3-75.2)	21.3% (16.2-27.6)	43.2% (36.3-50.5)
Specificity	58.0% (54.1-61.8)	90.5% (88.0-92.5)	79.1% (75.6-82.1)
Positive predictive value	33.2% (30.4-36.2)	40.4% (32.2-49.2)	38.4% (33.2-43.9)
Negative predictive value	86.1% (83.3-88.6)	79.2% (77.9-80.4)	82.2% (80.2-84.0)
Likelihood ratio for positive test	1.7 (1.4- 1.9)	2.3 (1.6-3.2)	2.1 (1.7-2.6)
Likelihood ratio for negative test	0.5 (0.4-0.7)	0.9 (0.8-0.9)	0.7 (0.6-0.8)
Pre-test probability of having a positive/non-conclusive exercise test	23.2%	23.2%	23.2%
Net gain after positive result	10.0% (7.2-13.3)	17.2% (9.0-26.0)	15.2% (10.0-20.7)
Number of patients with characteristic per positive/non-conclusive exercise test	3.0	2.5	2.6

*Chest pain when walking uphill or in a hurry. **Pathologic ST-T segment on resting electrocardiogram. ^†^Patient’s opinion of angina diagnosis: yes or no. CI: confidence interval. Positive exercise test: angina symptoms and ST-segment depression >0.1 mV during the test. Non-conclusive test: angina or ST depression. Negative test: neither angina nor ST depression.

**Table 5:** Composite diagnostic characteristics of exercise test outcome (positive/non-conclusive tests)

**Characteristic**	Exertional chest pain* and pathologic ST-T segment**	**Exertional chest pain and angina diagnosis according to patient’s opinion**^**†**^	**Exertional chest pain and pathologic ST-T segment and angina diagnosis according to patient’s opinion**
	**(95% CI)**	**(95% CI)**	**(95% CI)**
Number of patients with characteristic	26	74	15
Sensitivity	37.1% (26.8-48.9)	58.3% (49.6-66.5)	28.3% (18.0-41.6)
Specificity	92.5% (89.3-94.8)	78.5% (74.2-82.2)	96.6% (93.9-98.1)
Positive predictive value	59.9% (48.2-70.5)	45.0% (39.2-50.9)	71.5% (54.8-83.9)
Negative predictive value	83.0% (80.2-85.4)	86.2% (83.4-88.5)	81.7% (79.0-84.1)
Likelihood ratio for positive test	4.9 (3.1-7.9)	2.7 (2.1-3.4)	8.3 (4.0-18.0)
Likelihood ratio for negative test	0.7 (0.6-0.8)	0.5 (0.4-0.7)	0.7 (0.6-0.9)
Pre-test probability of having a positive/non-conclusive exercise test	23.2%	23.2%	23.2%
Net gain after positive result	36.7% (25.0-47.3)	21.8% (16.0-27.7)	48.3% (31.6-60.7)
Number of patients with characteristic per positive/non-conclusive exercise test	1.7	2.2	1.4

*Chest pain when walking uphill or in a hurry. **Pathologic ST-T segment on resting electrocardiogram. ^†^Patient’s opinion of angina diagnosis: yes or no. CI: confidence interval. Positive exercise test: angina symptoms and ST-segment depression >0.1 mV during the test. Non-conclusive test: angina or ST depression. Negative test: neither angina nor ST depression.

A theoretical simulation of receiver operating characteristic (ROC) curves from crude and adjusted ORs and a positive/non-conclusive exercise test as the dependent variable demonstrated an increasing area under the curve up to 0.82 for the combination of exertional chest pain, angina according to the patient, and ST-T changes in the composite model. The ROC curves and calculation are shown in Additional file 2. The multivariable model was also tested with men and women separately. For women, exertional chest pain (OR 1.98, 95% CI 1.15-3.43), angina according to the patient’s opinion (OR 1.86, 95% CI 1.04-3.34), and a pathologic ST-T segment on resting ECG (OR 2.82, 1.41-5.64) were independent predictors of exercise-testing outcome, as was systolic blood pressure (OR 1.02, 95% CI 1.00-1.03, p < 0.013). Among men, exertional chest pain (OR 3.11, 95% CI 1.83-5.29) and a pathologic ST-T segment on resting ECG (OR 2.05, 95% CI 1.08-3.86) remained significant. Angina according to the patient’s opinion was not significant in men only (OR 1.48, 95% CI 0.83-2.63). Age, dyslipidaemia, and previous cardiovascular events were not significant predictors of exercise-test outcome for men or women separately.

### Cardiovascular events

All patients were subject to follow-up for 180 days. Cardiovascular events occurred in 52.7%, 18.3%, and 2% of patients with positive, non-conclusive, and negative tests, respectively. The OR for any cardiovascular event was 54.6 (95% CI 25.5-117.0) for a positive test and 11.3 (95% CI 5.6-22.6) for a non-conclusive test versus a negative test. During follow-up, cardiovascular events occurred in 8% of all patients and 4% were referred for revascularization (Table 6). Of the 35 patients referred for revascularization, 18 underwent bypass grafting, and 17 received a percutaneous intervention. The predictive model classified 104 exercise tests as negative and 761 as positive. Sixty-seven out of 69 events (97.1%) occurred in patients with a predicted positive or inconclusive test, while two events (1.9%) occurred in patients with predicted negative tests (Table 6). These events were both cases of recurrent angina in primary care. The three cases of cardiovascular death all occurred among patients with a positive or non-conclusive exercise test. Nagelkerke R2 was 0.16. The Hosmer-Lemeshow test was not significant (p = 0.25) indicating good fit between data and model. No influential observations were identified through the Leverage statistic or DfBeta values. The bootstrapped standard errors were very close to the standard errors based on the entire sample.

**Table 6:** Cardiovascular events within 6 months according to observed and predicted outcome of exercise tests

**Type of cardiovascular event**	**Observed outcome of exercise test**				Predicted outcome of exercise test*		
	**Positive n=55**	**Non-conclusive n=142**	**Negative n=653**	**Non-assessable n=15**	**Positive/non-conclusive n=761**	**Negative/non-assessable n=104**	**Total n=865**
	**n (%)**	**n (%)**	**n (%)**	**n (%)**	**n (%)**	**n (%)**	**n (%)**
To GP for angina symptoms	13 (23.6%)	12 (8.5%)	11 (1.7%)	1 (6.7%)	35 (4.6%)	2 (1.9%)	37 (4.3%)
Hospitalized for unstable angina	10 (18.2%)	10 (7.0%)	2 (0.3%)	0	22 (2.9%)	0 (0%)	22 (2.5%)
STEMI/NSTEMI	1 (1.8%)	3 (2.1%)	0	0	4 (0.5%)	0 (0%)	4 (0.5%)
Revascularization	19 (34.5%)	12 (8.5%)	4 (0.6%)	0	35 (4.6%)	0 (0%)	35 (4.0%)
Cardiovascular death	1 (1.8%)	2 (1.4%)	0	0	3 (0.4%)	0 (0%)	3 (0.3%)
Patients with any event (%)	29 (52.7%)	26 (18.3%)	13 (2.0%)	1 (6.7%)	67 (8.8%)	2 (1.9%)	69 (8.0%)

GP: general practitioner. STEMI: ST-segment elevation myocardial infarction. NSTEMI: non-ST-segment elevation myocardial infarction.

Revascularization: decision on revascularization within six months. Cardiovascular death: cause of death was sudden cardiac death, myocardial infarction, or congestive heart failure. Positive exercise test: angina symptoms and depression of ST segment >0.1 mV during the test. Non-conclusive test: angina or ST depression. Negative test: neither angina nor ST depression. *Predicted outcome, classification cut off >0.1.

## Discussion

In this study, exertional chest pain, a pathologic ST-T segment on resting ECG, and angina diagnosis according to the patient’s opinion were independent predictors of exercise-test outcome. The observed OR for having any cardiovascular event within six months was 54.6 among patients with a positive exercise test and 11.3 among those with a non-conclusive test, compared to negative exercise tests. Patients with a negative exercise test had a very low risk of cardiovascular events. No patients predicted to have a negative exercise test by the multivariable model (>90% probability) were subject to hospitalization or death from coronary disease within six months.

Forty two percent of all patients with negative exercise tests reported chest pain on exertion (Table 2). Prevalent conditions among these patients were chronic pulmonary diseases, a clinical diagnosis of angina, anxiety disorder, non-specific chest pain, chest-wall pain, and upper gastrointestinal disorders. The diagnoses were not validated, but they offered an impression of the possible conditions associated with exertional chest pain that might result in a negative exercise test.

### Comparisons with previous research

The low incidence of cardiovascular events found in patients with a negative exercise test in the present study is comparable to findings by Sumanen et al. [35,36]; they found that the 2-year incidence of IHD in patients with negative exercise tests was 2% among middle-aged patients and 3% in older patients.

The review by Mant and colleagues stated that the exercise test is a relatively weak diagnostic test (LR + of 2.79 and likelihood ratio for negative test of 0.44 for 1 mm ST depression), and that results should not be interpreted isolated from the patient’s clinical history [37]. Our approach, which used exercise-test outcome as a proxy for coronary disease, is supported by the marked differences in the cardiovascular event rates experienced by our patients. A bivariate approach that compares positive and non-conclusive tests in one group with negative tests has similarities in clinical decision-making. Non-conclusive tests carry positive information in one of two possible respects, chest pain or ST-segment depression during exercise, which indicate coronary disease. Both positive and non-conclusive exercise tests need to be considered for clinical management.

The diagnostic importance of exertional angina is well established previously [5,25,38-42]. Exertional chest pain as an independent predictor in this study is therefore not surprising. The current investigation also considered the patient’s self-assessment of angina. A similar question was previously used in an investigation of the Marburg Heart Score [43], which expressed this factor as “patient assumes pain is of cardiac origin.” Neither age nor previous cardiovascular events were independent predictors in our study; this finding contrasted with the investigation based on the Marburg Heart Score. This might be explained by the different approaches used in these two studies. First, we recruited patients that had been referred to exercise testing due to a suspected IHD, not due to explicit chest pain symptoms. Thus, even patients with atypical symptoms were included in our cohort. Second, in the Marburg score analysis, age was dichotomized; in contrast, in our analysis, age was a continuous variable. Third, the resting ECG outcome was included in our model, but not in the Marburg score. The Marburg score has three modalities of chest pain: “Pain worse during exercise”, “pain not reproduced by palpation” and “patient assumes pain is of cardiac origin.” We did not include “pain upon palpation.” Our approach lacked an external expert panel as a reference standard; this approach simulated the primary care scenario. These methodological differences may have contributed to the differences between our results and those of the Marburg score analysis.

Our questionnaire required the patient to describe angina symptoms as “yes” or “no.” In the total adjusted model, the patient’s opinion was a significant predictor. In the regression analysis of men and women separately, the patient’s opinion was not a significant predictor in men (OR 1.48, 95% CI 0.83-2.63), but it was significant in women (OR 1.86, 95% CI 1.04-3.34). This observation requires explanation, but the impact of a smaller sample size is highlighted by widened confidence intervals.

The predictive importance of the resting ECG was addressed by Pryor et al. in 1993 [42] in a study of patients referred for a treadmill test. Q waves and ST-T segment changes predicted significant coronary artery disease on angiography and survival within three years in a multivariable regression model. A systematic review by Mant et al. in 2004 [37] identified 13 relevant studies of the use of resting ECGs in the diagnosis of IHD in primary-care patients with suspected exertional angina. The review concluded that a resting ECG was only of limited value for evaluating patients with suspected angina. ST- and T-wave changes were not useful, and neither was a normal resting ECG. Q waves were the most frequently reported ECG change, within a wide confidence interval (LR+, OR 2.56, 95% CI 0.89-7.30) [37]. In our study, a pathologic ST-T segment on resting ECG was a significant predictor of exercise-test outcome in the total model and for women and men separately. Q waves on resting ECG were not helpful predictors in our study. Comparing our findings with previous reports on resting ECG as a diagnostic tool is not straightforward; in our study, we predict the outcome of exercise tests, not IHD proven by angiography. We believe that the potential utility of a resting ECG as a predictor of coronary disease is dependent on which type of reference standard is chosen.

### Cardiovascular events and predictive model

A delayed-type reference standard has been used in several previous studies of chest pain and IHD in primary care [8,10,24,26,41,44] because validation by coronary angiography is not justified in a setting with a low disease prevalence. Use of the clinical course as a reference standard is achievable in primary-care practice and is not subject to bias, as long as follow-up is complete for all patients. We found that patients with a negative exercise test had a low cardiovascular risk within 180 days. Of 35 patients with revascularization, 31 were recruited from the 197 patients with a positive or non-conclusive test and only four patients were derived from the population of 653 patients with a negative exercise test.

Among patients classified as negative by the multivariable regression model (104/865), no cases with hospitalization for coronary disease or cardiovascular death occurred within six months. The probability cut off of >10% for an exercise test to be classified as positive/non-conclusive was chosen from a clinical standpoint. A prediction model designed to rule out disease must be evaluated against the number of false-negative cases to be clinically useful.

### Strengths and limitations

Enrolment occurred in a clinical setting representing the normal care of patients who consulted GPs for suspected angina symptoms. Registration of cardiovascular events was completed without losses to follow up. Background variables contained few missing data and all exercise tests were performed by the same laboratory. The cardiovascular events during the follow-up were recorded from medical records, scrutinized by GN (author) and one assistant. A blinding to the outcome of exercise tests was not possible, because the records were complete, with no pre-selection of data.

We did not use coronary angiography for verification of disease, which is often standard. In primary care with predominantly low-risk patients, coronary angiography would not be justified in all cases, and we sought to study all patients referred to exercise tests for a suspicion of IHD. Registration of previous cardiovascular morbidity from a questionnaire is not faultless, but neither are medical record data. The use of self-reported history of IHD was supported by previous research [45]. Medication was registered from questionnaires, since medication lists sometimes are not up to date and patient compliance toward a medication list is not always complete. Physicians conducting the ECG readings were not blinded to the patient; blinded reading would be preferable in a study that specifically aims to explore the potential of resting ECG to predict exercise-test outcome.

Nearly half of the population was living in rural municipalities, and the average educational level was low. The access to primary health care was equivalent among the rural municipalities, compared to the central municipality of Östersund. Long distances to the central hospital might have been a problem for patients living in remote areas. Our results should be evaluated in relation to the availability of health care and baseline demographics in other populations.

For internal validation, we applied a bootstrapping procedure. An alternative approach would have been to divide the cohort in two to create a derivation cohort and a validation cohort. This was considered, but not applied, because it would have resulted in a loss of statistical power compared to the bootstrapping procedure. Due to this limitation, the clinical characteristics and the multivariate model described in this study should be validated in an external primary-care cohort.

### Clinical importance

We developed a clinical prediction model to characterize patients before exercise testing. We consider our results to be representative for patients of similar backgrounds living in countries with similar access to health care.

## Conclusions

Clinical characteristics can be used to predict the outcome of exercise testing. The outcome of clinical exercise tests in primary-care patients predicts the risk of cardiovascular events; patients with a negative test have a very low risk of cardiovascular events within six months. A predictive model of exercise-test outcome, based on clinical characteristics, can be used to refine the identification of low-risk patients.

## Competing interests

The authors declare that they have no competing interests. The study was sponsored by a grant from AstraZeneca (50 000 SEK in 2010). AstraZeneca had no input in study design, data interpretation, or writing of the manuscript.

## Authors’ contributions

GN conceived the study design, performed the statistical analysis, and drafted the manuscript. ES supervised the study, participated in the study design and analysis of data, and helped to draft the manuscript. TM supervised the study, participated in the study design and analysis of data, and helped to draft the manuscript. HS: gave advice for the statistical analysis and evaluated the internal validity of the multivariable regression model. All authors read and approved the final manuscript.

## Authors’ information

GN: GP, Doctoral student, Family medicine, Department of Public Health and Clinical Medicine, Umeå University, Umeå, Sweden (Jämtland Cty Council, Research Unit, SE-83157 Östersund, Sweden).

ES: GP, Associate Professor of Family medicine, Department of Public Health and Clinical Medicine, Umeå University, Umeå, Sweden.

TM: Consultant in cardiology, Östersund Hospital, Associate Professor of Medicine, Department of Public Health and Clinical Medicine, Umeå University, Umeå, Sweden.

HS: Senior lecturer, Epidemiology and Global Health, Department of Public Health and Clinical Medicine, Umeå University, Umeå, Sweden.

## Pre-publication history

The pre-publication history for this paper can be accessed here:

http://www.biomedcentral.com/1471-2296/15/71/prepub

## Supplementary Material

Additional file 1 **Resting ECG criteria for myocardial scarring and a pathologic ST-T segment.** Click here for file

Additional file 2 Theoretical simulation of receiver operating characteristic (ROC) curves from crude odds ratio (OR; A) and from combinations of adjusted OR (B). Click here for file
