# Supplementary material for: Diagnostic characteristics and prognoses of primary-care patients referred for clinical exercise testing: a prospective observational study
Source: BMC Fam Pract. 2014 Apr 18;15:71. doi: 10.1186/1471-2296-15-71 (PMC4021414; doi:10.1186/1471-2296-15-71)
Supplement: Additional file 1 — Resting ECG criteria for myocardial scarring and a pathologic ST-T segment. [file 1471-2296-15-71-S1.pdf]

Additional data, file 1 Resting ECG criteria for myocardial scarring and a pathologic ST-T segment

Myocardial scarring: pathological Q-wave or altered R-wave progression compared to a previous ECG.

Pathological Q wave (Minnesota Code I, 1): “Q and QS items, (any of the items described in **a** through **g**):

- **a.** Q/R=1/3 or more and Q duration=0.03 s or more, in any of leads I, II, or V2-V6;
- **b.** Q duration=0.04 s or more, in any of leads I, II, or V1-V6;
- **c.** Q duration=0.04 s or more and R amplitude 3 mm or more, lead aVL;
- **d.** Q duration=0.05 s or more and a Q wave present in leads aVF or III;
- **e.** Q duration=0.05 s or more, lead aVF;
- **f.** QS pattern, when R wave is present in adjacent precordial lead to the right, in any of leads V2-V6;
- **g.** QS pattern in leads V1-V4; V1-V5; or V1-V6.”

Pathological ST-segment and T-wave items: (Minnesota Code IV, 1-3 and Code V, 1-3): “S-T junction and segment (Measured from preceding P-R interval at onset of QRS) Depression:

1. S-T junction depression of 1 mm or more in any of leads I, II, aVL, aVF, or V1-V6;
2. S-T junction depression 0.5-0.9 mm and S-T segment horizontal or downward sloping, in any of leads I, II, aVL, aVF, or V1-V6;
3. No S-T junction depression of at least 0.5 mm but an S-T segment sloping down to reach at least 0.5 mm below the P-R baseline in any of leads I, II, aVL, aVF, or V1-V6.”

“T-wave items:

1. T-amplitude of minus 5 mm or below in any of leads I, II, or V2-V6; when the R amplitude=5 mm or more in lead aVL; when the QRS is mainly upright in lead aVF;
2. T-amplitude of minus 1 to 5 mm in any of leads I, II, or V2-V6; when the R amplitude=5 mm or more in lead aVL; when the QRS is mainly upright in lead aVF;
3. T wave is flat or slightly diphasic (negative phase less than 1 mm) in any of leads I, II, or V3-V6; when the R amplitude=5 mm or more in aVL; when the QRS is mainly upright in lead aVF.”
