# Supplementary material for: Diagnostic characteristics and prognoses of primary-care patients referred for clinical exercise testing: a prospective observational study
Source: BMC Fam Pract. 2014 Apr 18;15:71. doi: 10.1186/1471-2296-15-71 (PMC4021414; doi:10.1186/1471-2296-15-71)
Supplement: Additional file 2 — Theoretical simulation of receiver operating characteristic (ROC) curves from crude odds ratio (OR; A) and from combinations of adjusted OR (B). [file 1471-2296-15-71-S2.pdf]

A) B)

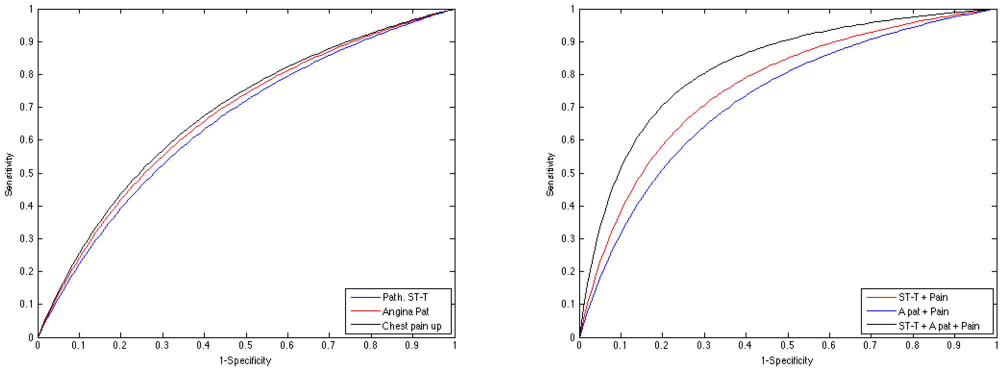

Additional data, file 2 Theoretical simulation of receiver operating characteristic (ROC) curves from crude odds ratio (OR; A) and from combinations of adjusted OR (B)

Theoretical estimation of the area under the curve from crude OR

Pathologic ST-T segment on resting ECG:	0.6534
Angina according to patient:	0.6700
Chest pain on exertion	0.6804

Theoretical estimation of the area under the curve from combinations of adjusted OR

Pathologic ST-T + Chest pain on exertion:	0.7622
Angina according to patient+ Chest pain on exertion:	0.7233
Pathologic ST-T + Angina according to patient + Chest pain on exertion:	0.8225

Simulation of ROC curve and estimation of the area under the curve

An ROC curve with the corresponding area under the curve can be simulated by using the relations in a standard four-way table (Additional Table 1) for dichotomous evaluation of a marker compared to a clinical outcome. Using Table 1, the OR is estimated as $\frac{a*d}{b*c}$.

Additional Table 1 Standard presentation of the outcome from a marker compared to clinical outcome

		Result of marker	
		+	-
Clinical outcome	+	a	b
	-	c	d

Using the sensitivity, $\frac{a}{a+b}$, and the specificity, $\frac{d}{c+d}$, the OR can also be expressed as OR = $\frac{Sens/(1-Sens)}{(1-Spec)/Spec}$. To simplify the equation, let y = sensitivity, x = specificity, and μ = OR. The relation between OR and sensitivity and specificity can then be expressed as $\frac{y/(1-y)}{(1-x)/x}$ = μ, which yields $\frac{y}{1-y} = \frac{x}{1-x} * \mu$.

The sensitivity, y, can now be expressed as y = $\frac{\mu x}{\mu x - x + 1}$ or as

Sens = $\frac{OR*Spec}{OR*Spec - Spec + 1}$.

This implies that for a fixed OR, the sensitivity can be estimated for any given value of the specificity, which means that a ROC curve can be constructed.

The area under the curve (AUC) can be estimated by integrating the expression above, given that ln(OR) > 0:

$$\text{AUC} = \int_0^1 y \, dx = \int_0^1 \frac{\mu x}{\mu x - x + 1} \, dx = \ldots = \frac{\mu(\mu - 1 - \ln(\mu))}{(\mu - 1)^2}$$

if ln(OR) ~ 0 => AUC ~ 0.5.
